# Early Congenital Syphilis Presenting With Severe Congenital Pneumonia and Cutaneous Manifestations in a Neonate at Birth: A Case Report

**DOI:** 10.7759/cureus.69849

**Published:** 2024-09-21

**Authors:** Mohammed H Alqahtani, Faisal S Alanazi, Hassan S Alqahtani, Abdulrahman S Altowaim, Ibrahim A Alanzi

**Affiliations:** 1 Neonatology, Al Yamamah Hospital, Riyadh, SAU; 2 Pediatrics Endocrinology, Al Kharj Military Industries Corporation Hospital, Al Kharj, SAU; 3 College of Medicine, King Saud Bin Abdulaziz University for Health Sciences, Riyadh, SAU

**Keywords:** congenital syphilis, neonatal infection, pediatric dermatology, pneumonia alba, treponema pallidum

## Abstract

Transplacental transmission of *Treponema pallidum* spirochetes from an infected mother to the fetus during pregnancy results in the infectious condition known as congenital syphilis (CS). Once a forgotten disease, CS has now re-emerged. We report the clinical case of an early CS in a neonate girl presented with severe congenital pneumonia (pneumonia alba) requiring intubation, along with skin lesions that were visible from birth on the palms and soles of the feet. Neonate’s treponemal and non-treponemal tests were positive, and the mother’s rapid plasma reagin (RPR) test was also positive after birth, and the mother did not have prenatal screening or follow-ups due to socioeconomic reasons. Neonate’s radiological investigations found a diffuse, uniform opacification of both lungs ("white lung") on chest X-ray, consistent with congenital pneumonia (pneumonia alba), in addition to increased radiolucency, widening, irregularity, and erosions in the lower limbs, with periventricular leukomalacia and possible petechial hemorrhages on cerebral magnetic resonance imaging. The neonate was given a 10-day course of intravenous penicillin G along with five days of concurrent inotrope support, IV hydrocortisone for seven days, IV vancomycin and meropenem for 10 days, along with respiratory support. The neonate stayed in the neonatal intensive care unit (NICU) for a period of 54 days in total and was discharged with a clinically favorable outcome.

## Introduction

Congenital syphilis (CS) results from the transmission of the bacterium *Treponema pallidum* from the infected mother to the fetus [1]. Only severe infections are observable after birth, while other results can be stillbirth, preterm births, or a wide array of clinical symptoms [2]. It is anticipated that CS affects around one million pregnancies annually across the globe and remains one of the significant public health burdens [3]. The exact incidence of CS cannot be accurately obtained; however, from the available data, it clearly appears that over the last 10 years, the disease has become significantly more common in the United States [4]. Herein, we present a case of a neonate presented at birth with severe congenital pneumonia and skin lesions of early CS.

## Case presentation

We report a case of a neonatal girl born with severe congenital pneumonia (pneumonia alba) presenting in severe respiratory distress and grunting; cutaneous lesions on palms and soles were noted at birth. The mother was a 19-year-old Gravida 1 Para 0 who received unsatisfactory prenatal care due to socioeconomic circumstances. Parents were sexually active and were not known to have had multiple sexual partners. They were not consanguineous; the mother was asymptomatic and healthy, but the father had small painless sores on his penis, and he received a local application. The neonate was born at 37 weeks, a product of a normal spontaneous vaginal delivery (NSVD) with intrauterine growth restriction (IUGR) with meconium-stained amniotic fluid (MSAF). Apgar scores were 7 at one minute and 8 at five minutes; the baby cried but weakly and needed initial steps with suction that showed thin meconium and also required positive pressure ventilation (PPV) and then intubation. Birth weight was 1.8 kg (<3rd percentile), length was 40.5 cm (<3rd percentile), and head circumference was 31 cm (<2.5th percentile).

**Figure 1:**
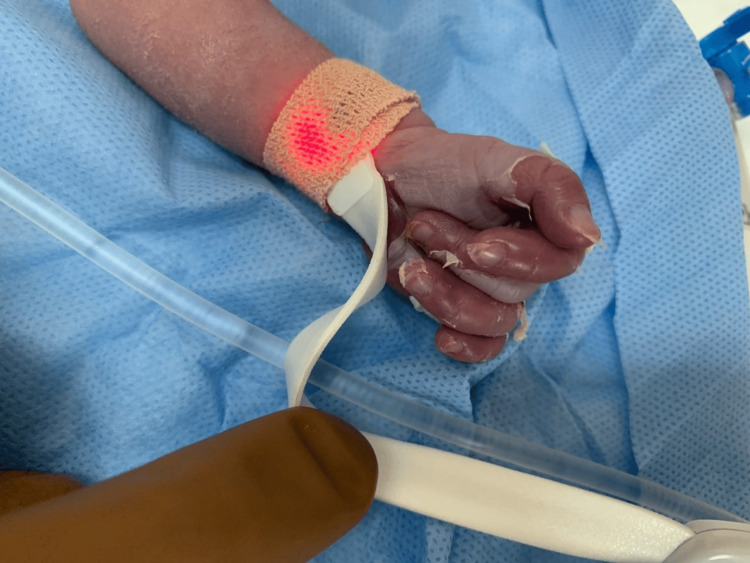
Pemphigus syphiliticus of the hand which was present at birth The image shows the neonate's hand with erythematous skin, mild edema, and prominent desquamation of the fingers, consistent with syphilitic pemphigus.

**Figure 2:**
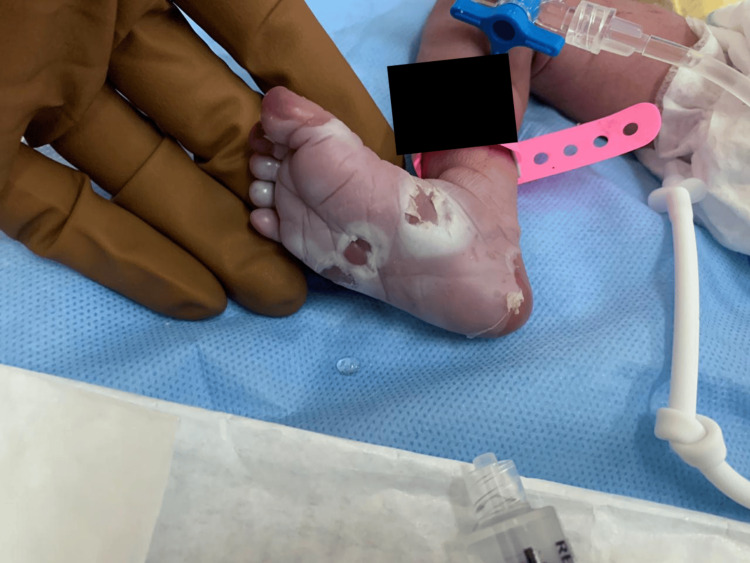
Peeled blister lesions indicative of pemphigus syphiliticus on neonatal foot (plantar surface of the right foot) This is the foot of the neonate with numerous skin lesions characteristic of pemphigus syphiliticus. The lesions are in the form of superficial erosions and blisters in irregular outlines, some of which have burst to leave raw, eroded surfaces. The surrounding skin is erythematous and slightly edematous, consistent with an inflammatory condition. Most often, these lesions are a presentation in early congenital syphilis and demand immediate medical attention and care to prevent further complications in eradicating the infection of syphilis.

**Figure 3:**
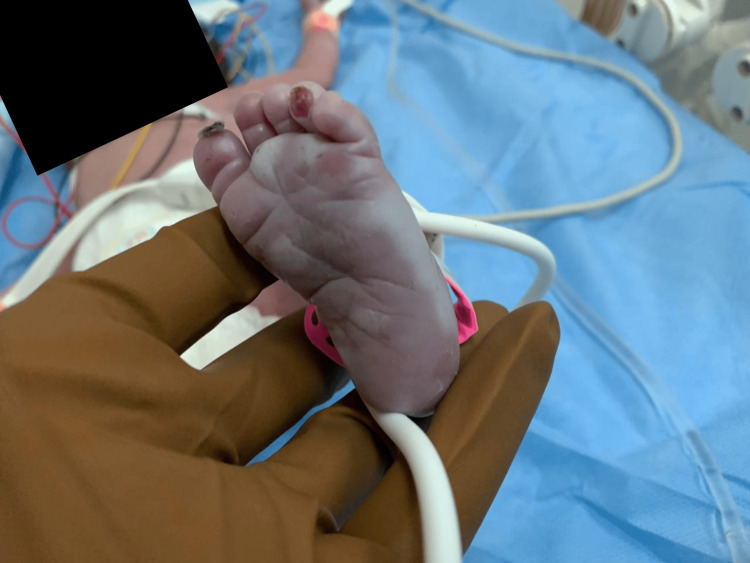
Crusted and erythematous lesions on the toes of the neonate with syphilitic pemphigus (plantar surface of the left foot) The image shows a desquamating lesion on the big toe, a crusted lesion on the tip of the second toe, and erythematous skin on the foot of a neonate. These characteristics point to pemphigus syphiliticus, the pathognomonic lesion for congenital syphilis.

On clinical examination, she demonstrated normal muscular tone, no neurological impairments, and no dysmorphic characteristics. Skin examination revealed bullous eruption (pemphigus syphilitics) on the palms and soles (Figures 1-3). The chest exam revealed tachypnea, chest retractions, decreased breath sounds, crackles, cyanosis, grunting, and glue-like secretions. The abdomen was moderately distended. Other systemic examinations were unremarkable. Last, the hearing assessment was normal.

A complete blood cell count demonstrated a hemoglobin of 11.4 grams per deciliter, a white blood cell count of 29,000 per cubic millimeter, and a platelet count of 156,000 per cubic millimeter. The Coombs test was negative. The serum total protein was 62 grams per deciliter with an albumin level of 14 grams per deciliter. C-reactive protein (CRP) was 12.8 milligrams per liter. Also, the aspartate aminotransferase (AST) level was twice the normal value, the alanine aminotransferase (ALT) level was normal, and the total bilirubin level was 75 milligrams per deciliter. Serologic tests for TORCH (including toxoplasmosis, syphilis, rubella, cytomegalovirus, and herpes simplex) were all negative except for reactive results of *T. pallidum* hemagglutination (TPHA) and rapid plasma reagin (RPR) for syphilis (Table 1).

**Table 1:** Lab findings WBC: white blood cells; RBC: red blood cells; HGB: hemoglobin; HCT: hematocrit; MCV: mean corpuscular volume; MCH: mean corpuscular hemoglobin; MCHC: mean corpuscular hemoglobin concentration; RDW-CV: red cell distribution width-coefficient of variation; PLT: platelets; NEUTS %: neutrophils percentage; LYMPH %: lymphocytes percentage; MONO %: monocytes percentage; EOS %: eosinophils percentage; BASO %: basophils percentage; NEUT: neutrophils; LYMPH: lymphocytes; MONOCYTES: monocytes; EO: eosinophils; BASO: basophils; CRP: C-reactive protein; AST: aspartate aminotransferase; ALT: alanine aminotransferase

Test name	Result	Unit	Normal range
WBC	29	x10^3/µL	10-26
HGB	11.4	g/dL	14-22
HCT	35.1	%	45-75
MCV	109	FL	100-120
MCH	35.4	PG	31-37
MCHC	32.5	g/dL	30-36
RDW-CV	20.6	%	11-14
PLT	156	x10^3/µL	100-450
Reticulocyte	5	%	0.5-1.5
NEUTS %	58.2	%	40-55
LYMPH %	30.7	%	30-45
MONO %	9.2	%	0-9
EOS %	1.1	%	1-12
BASO %	0.8	%	0.5-1
NEUT	16.88	x10^3/µL	4-14
LYMPH	8.9	x10^3/µL	3-8
MONOCYTES	2.66	x10^3/µL	0.5-2
EO	0.32	x10^3/µL	0.1-1
BASO	0.24	x10^3/µL	0.05-0.15
Serum total protein	62	g/dL	45-75
Albumin	14	g/dL	28-44
CRP	12.8	mg/L	<10
AST	144	U/L	20-80
ALT	27	U/L	5-45
Total bilirubin	75	mg/dL	1-10

The neonate’s RPR and venereal disease research laboratory test (VDRL) were positive, and the TPHA titer was positive at 1:160. The mother had a positive RPR test after birth, and she had not been screened before for syphilis. The neonate’s cerebrospinal fluid (CSF) examination was normal, and the CSF venereal disease research laboratory test (CSF VDRL) was non-reactive. In the cultures of blood, urine, or CSF, no microbes were found. The CSF was colorless and clear, with no white blood cells and 240 red blood cells per cubic millimeter. The CSF glucose level was 4.1 mmol/L (high abnormal), and the CSF protein level was 93.1 mg/dL (high abnormal). No organisms were seen on Gram stain (Table 2).

**Table 2:** Cerebrospinal fluid profile WBC: white blood cells; RBC: red blood cells; CSF: cerebrospinal fluid

Test name	Result	Unit	Normal range
Color	Colorless		
Appearance	Clear		
WBC	NIL	WBC/mm³	
RBC	240	RBC/mm^3^	
Gram stain	No organism seen		
CSF glucose	4.1	mmol/L	2.2-3.9
CSF protein	93.1	mg/dL	15-45

**Figure 4:**
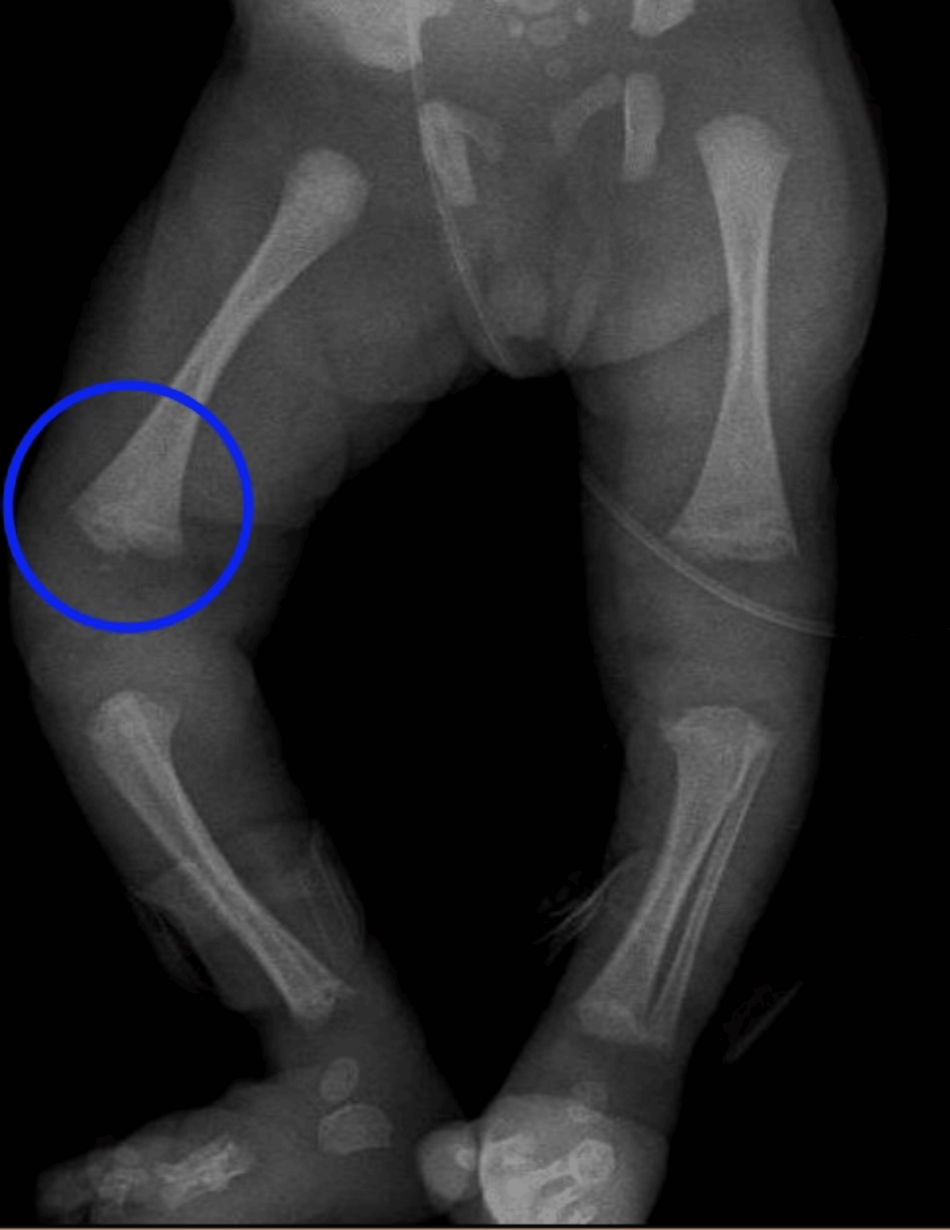
An anteroposterior (AP) X-ray An anteroposterior (AP) X-ray shows increased radiolucency, widening, irregularity, and erosions in the metaphyseal regions of the long bones, with the distal regions affected more than the proximal, and these findings are more obvious in the right lower limb (blue circle).

**Figure 5:**
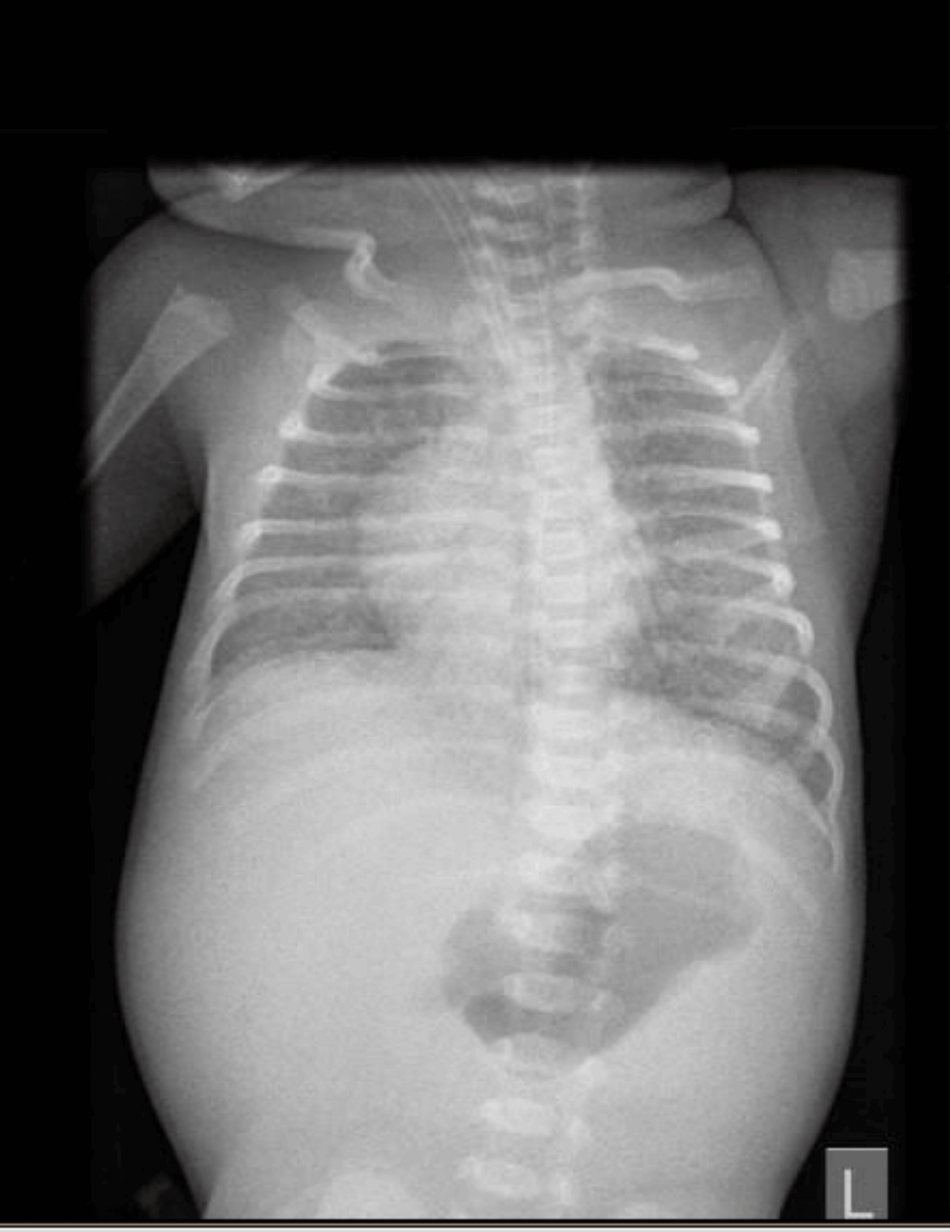
Frontal projection chest X-ray Frontal projection chest X-ray shows a diffuse bilateral pulmonary opacification giving a characteristic "white lung" appearance due to extensive consolidation which is consistent with pneumonia alba.

A long bone X-ray revealed increased radiolucency, widening, irregularity, and erosions in the metaphyseal regions of the long bones, more pronounced in the lower limbs, particularly on the right side (Figure 4). Chest X-ray showed a diffuse, uniform opacification of both lungs ("white lung") consisting of congenital pneumonia (pneumonia alba), distinguished from other neonatal conditions by the presence of air bronchograms, indicating air-filled bronchi against the surrounding consolidated lung tissue (Figure 5). The cerebral magnetic resonance imaging report was consistent with periventricular leukomalacia changes and possible petechial hemorrhages or calcifications.

**Figure 6:**
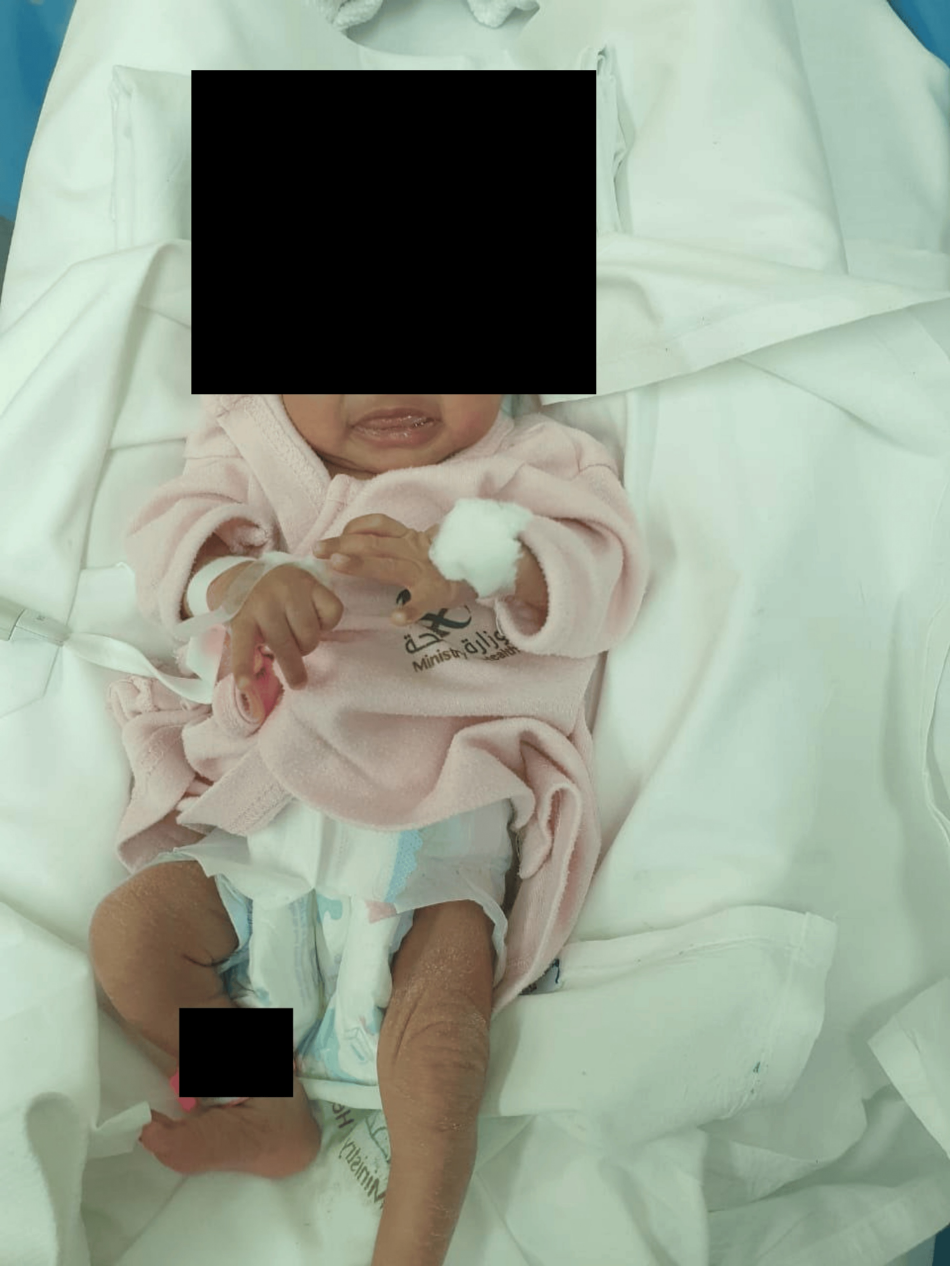
The infant after 54 days at neonatal intensive care unit The image shows the infant after treatment for early congenital syphilis, showing clear skin, normal facial features, and no visible signs of previous symptoms, indicating a successful resolution of the disease.

For the treatment of CS, 50,000 units/kg body weight/dose IV penicillin G was administered during the first seven days of life every 12 hours, then every eight hours thereafter to complete a total of 10 days. Despite initial PPV via a T-piece, the neonate’s respiratory distress persisted, with SpO_2_ levels between 43% and 50%, grunting, and a dusky appearance. Endotracheal intubation was performed, and the infant was placed on high-frequency oscillatory ventilation (HFOV) with a mean airway pressure (MAP) of 20 cmH_2_O, a pressure amplitude (P) of 40 cmH_2_O, and a fraction of inspired oxygen (FiO_2_) of 100%. To support cardiac function, the neonate received inotropes, including dopamine at 5 mcg/kg/min and dobutamine at 10 mcg/kg/min, administered continuously for five days. Moreover, the newborn received additional treatment with IV hydrocortisone at 1.5 mg/kg every eight hours for seven days, IV vancomycin at 18 mg/kg every 12 hours for 10 days, and IV meropenem at 36 mg/kg every eight hours for 10 days. The neonate remained in the NICU for 54 days due to ongoing respiratory distress, treatment for CS, and close monitoring for complications such as periventricular leukomalacia (PVL) and IUGR. The patient was eventually discharged in stable condition, having successfully weaned off respiratory support and tolerating oral feeding, with a noticeable resolution of the dermatological signs of syphilis before discharge (Figure 6).

## Discussion

CS is an infection caused by the spirochete bacteria *T. pallidum, *which is highly infectious; it is transmitted from an infected mother to the fetus during pregnancy through the placenta [5]. This transmission may occur at any stage of pregnancy, but the risk is particularly high in cases of untreated primary or secondary syphilis, where transmission is highly likely during the third trimester [1]. CS can cause a generalized infection that affects virtually all body systems and tissues, with the skin, mucous membranes, bones, liver, spleen, and central nervous system being the most affected [6].

CS is indeed a significant public health concern. The latest epidemiological report for 2022, published by the European Centre for Disease Prevention and Control (ECDC), revealed an increase in the number of CS cases reported in the European Union/European Economic Area (EU/EEA) in 2022 and 2021, following a decline in notifications during 2020 [7]. In the US, studies showed a significant increase in the incidence of syphilis and CS since 2012. In the year 2020, the Centers for Disease Control and Prevention (CDC) reported a national record of 2,148 cases of CS [4].

CS is classified into early CS (clinical manifestations up to two years of age) and late CS (clinical manifestations in children older than two years), and the infection can be acquired during any trimester of pregnancy [8]. Only a few CS cases are symptomatic, while about two-thirds of the cases may remain asymptomatic at birth. If left untreated, clinical symptoms typically develop between 5 and 12 weeks of age [9]. The typical classic symptoms of early CS include fever, laryngitis, hemolytic anemia, glomerulonephritis, petechiae, swelling of lips, blisters, giant condylomas, mucosal ulcers, rhinitis, pseudoparalysis, interstitial hepatitis, lymphadenopathy, enteritis resistant to treatment, and blisters. Symptoms of meningitis typically become apparent within three to six months of age, although other forms of CNS injuries, such as hydrocephalus and convulsions, may also manifest [6]. However, the most dramatic physical finding in our case was respiratory distress, a rare occurrence in CS. This symptom may be attributed to pneumonia alba, where lung tissue becomes pale and firm due to inflammation and fibrosis. This term was first coined by Rudolf Virchow in 1866, based on autopsy findings of affected infants [10]. CS that is left untreated for an extended period of time can cause permanent abnormalities such as neurological deafness, interstitial keratitis, and the distinctive Hutchinson's teeth [11].

Diagnostic testing for CS is essential for newborns whose mothers tested positive for syphilis during pregnancy [12]. The process begins with a meticulous physical examination to get signs of CS. Also, quantitative nontreponemal testing, including the VDRL or RPR test, should then be conducted in the serum of the newborn, using the same type of test as that obtained on the mother to enable an exact comparison of titers. Other investigations for *T. pallidum* should also be considered when suspicious lesions or nasal discharge are present, such as direct fluorescent antibody (DFA) staining, darkfield microscopy, or PCR. On the other hand, pathological examination of the placenta or the umbilical cord can also be done when possible [8]. During a lumbar puncture (LP), a CSF examination should include a VDRL test, assessments of CSF protein and glucose, and a cell count with differential. In certain cases, this technique can help diagnose CS in infants and determine whether continuous CSF monitoring is required [13].

The diagnosis in our patient was established based on clinical manifestations of early CS, such as pemphigus syphilitics on the palms and soles, hemolytic anemia, IUGR, and congenital pneumonia (pneumonia alba), which were present at birth. Radiological investigations found increased radiolucency, widening, irregularity, and erosions in the lower limbs with periventricular leukomalacia and possible petechial hemorrhages on cerebral magnetic resonance imaging. The diagnosis was reinforced by the positivity of the neonate’s RPR and VDRL; TPHA test titer was positive at 1:160. Additionally, the mother had a positive RPR test for the first time after birth, as the mother did not have prenatal screening for infectious diseases or even a proper follow-up.

The management of CS has become standardized according to the neonate's category based on the likelihood of infection [1]. In cases where a mother's diagnosis of syphilis during pregnancy results in reactive nontreponemal and treponemal tests, regardless of treatment, and the newborn exhibits any of the following symptoms: clinical signs consistent with syphilis congenital; a serum VDRL or RPR titer in the infant that is at least four times higher than the mother's; positive results from darkfield microscopy or PCR of skin lesions, body fluids, placenta, or umbilical cord; or abnormal results from LP, such as CSF cell count, protein, and VDRL, necessitates further assessment and management. This is indeed the condition under which a 10-day course of IV aqueous crystalline penicillin G is recommended, as was in our case. All infants and children with reactive serologic tests for syphilis or who are born to mothers who were seroreactive at delivery should be followed up with examinations to ensure they are responding as expected to treatment [12].

## Conclusions

CS is far from eradicated; in fact, there is an observed increase in its incidence and a reemergence as a public health issue worldwide, including in developed countries. Despite being a rare disease with straightforward prevention, CS remains a significant source of perinatal and neonatal morbidity and mortality. Through this case, we highlight the importance of recognizing the clinical manifestations of early CS to diagnose and treat newborns promptly, thereby preventing severe consequences and prolonged hospitalizations.
